# Biofilms in hoses utilized to divert colostrum and milk on dairy farms: A report exploring their potential role in herd health, milk quality, and public health

**DOI:** 10.3389/fvets.2022.969455

**Published:** 2022-08-26

**Authors:** Alejandra A. Latorre, Ricardo Oliva, Julio Pugin, Alexis Estay, Francisco Nualart, Katterine Salazar, Natacha Garrido, Marcos A. Muñoz

**Affiliations:** ^1^Departamento de Patología y Medicina Preventiva, Facultad de Ciencias Veterinarias, Universidad de Concepción, Chillán, Chile; ^2^Centro de Espectroscopía y Microscopía Electrónica, Universidad de Concepción, Concepción, Chile; ^3^Departamento de Biología Célular, Facultad de Ciencias Biológicas, Centro de Microscopía Avanzada, Universidad de Concepción, Concepción, Chile; ^4^Hospital Dr. Víctor Ríos, Servicio de Salud Bío Bío, Los Ángeles, Chile; ^5^Departamento de Ciencia Animal, Facultad de Ciencias Veterinarias, Universidad de Concepción, Chillán, Chile

**Keywords:** biofilms, milking equipment, milk hoses, colostrum, diverted milk

## Abstract

Biofilms in milking equipment on dairy farms have been associated with failures in cleaning and sanitizing protocols. These biofilms on milking equipment can be a source of contamination for bulk tank milk and a concern for animal and public health, as biofilms can become on-farm reservoirs for pathogenic bacteria that cause disease in cows and humans. This report describes a cross-sectional study on 3 dairy farms, where hoses used to divert waste milk, transition milk, and colostrum were analyzed by culture methods and matrix-assisted laser desorption/ionization time-of-flight mass spectrometry (MALDI-TOF MS) to assess the presence of pathogenic bacteria such as *Staphylococcus aureus, Pseudomonas aeruginosa*, and *Klebsiella* spp. In addition, the presence of biofilms was analyzed using scanning electron microscopy and confocal spectral microscopy. Biofilms composed of multispecies microbial communities were observed on the surfaces of all milk hoses. In two dairy farms, *S. aureus, P. aeruginosa, Klebsiella pneumoniae*, and *Klebsiella oxytoca* were isolated from the milk hose samples collected. Cleaning and sanitation protocols of all surfaces in contact with milk or colostrum are crucial. Hoses used to collect waste milk, colostrum, and transition milk can be a source of biofilms and hence pathogenic bacteria. Waste milk used to feed calves can constitute a biosecurity issue and a source of pathogens, therefore an increased exposure and threat for the whole herd health and, potentially, for human health.

## Introduction

Biofilms are microbial communities attached to surfaces by means of an exopolymeric matrix (1). This matrix is made of several substances, including polysaccharides, proteins, DNA, and other matrix-entrapped environmental substrates (2, 3). The presence of biofilms has been previously documented on dairy farms, including on-farm cooling systems (4), water troughs (5), milk tank, and milking equipment (6–9).

Several microorganisms known for their ability to form biofilms, such as *Pseudomonas* spp., *Staphylococcus aureus, Listeria monocytogenes* strains, *Bacillus* strains, and *Klebsiella* spp. have often been found on dairy farms or in bulk tank milk (10–15). In addition, pathogenic microorganisms such as *S. aureus* have the ability to adhere to materials that are often used for the manufacture of milking equipment parts, such as stainless steel and rubber (9, 16, 17). Microorganisms such as *Pseudomonas* spp., which can have increased ability to produce exopolymeric matrix (18), have also been reported on dairy farms in places such as teat cups, drains, water, and sand used for bedding (19, 20). Microorganisms in biofilms usually live within microbial communities formed by several species (7, 21), where the detachment of bacteria to the environment can be used as a dispersal strategy (21). In the case of biofilms on milking equipment surfaces, because bacteria can be dislodged by the milk flow during milking (6, 17) the biofilm's microbiota can be transported to other locations within the milking system or the milk tank. These events are relevant to milk quality and food hygiene, as bacteria from biofilms in milking equipment can not only cause contamination of bulk tank milk (7, 9, 17), but also colonize and persist for long periods of time within the milking system (15). A recent study (9) also shows that biofilms in rubber liners on milking machine claws can be a source of *S. aureus* for the udder, thus posing a risk of intramammary infections due to the close contact of biofilm-fouled liners with the teat during milking.

In dairy operations around the world, colostrum and transition milk, milk from cows with clinical mastitis, cows undergoing antibiotic or anti-inflammatory treatments, or intramammary-infected high somatic-cell-count cows are usually diverted from the bulk tank, as required by law or by the milk processors. Likewise, in many places of the world, it is a common practice to segregate clinical mastitis and treatment cows, as well as fresh cows, to milk them at the end of the milking routine. Thus, their milk, colostrum, or transition milk is collected using milk cans. Standard cleaning and sanitation protocols for these milk cans, hoses, and other parts of milking equipment used to harvest waste milk/colostrum are, however, often neglected. The presence of milk and colostrum residues in poorly cleaned or non-cleaned milking equipment provides not only a source of nutrients for the proliferation of microorganisms (22), but also a “conditioning film” (23) for surfaces. These factors can create ideal conditions for bacteria colonization and the subsequent biofilm formation in such areas of the milking equipment, where cleaning and sanitation protocols may not have been properly conducted. If formed, these overlooked biofilms may pose a threat for both animal and public health, as they can become on-farm reservoirs for pathogenic bacteria known to cause disease in cows and humans (7, 9).

This report describes the findings on 3 Chilean dairy farms, where pieces of milking equipment used to collect waste milk, transition milk, and colostrum were removed and analyzed to assess the presence of biofilms and pathogenic bacteria such as *S. aureus, P. aeruginosa*, and *Klebsiella* spp. These three organisms were sought due to both their putative high potential to form biofilms (24–29) and their documented importance in animal and human health. To our knowledge, this is the first report assessing the role of biofilms in hoses used for waste milk, and the relevance of neglecting parts of milking equipment used for purposes other than collecting bulk tank milk.

## Methods

### Study farms

A cross-sectional study was conducted on three dairy farms of the Ñuble Region of Chile. The three farms, namely Farm A, Farm B, and Farm C had 74, 170, and 46 lactating cows, respectively. Farm A had a year-round grazing system, whereas Farms B and C had a free-stall seasonal confinement system. All farms had automated milking equipment and a bulk milk tank for cooling and storage of salable milk.

Washing and sanitation procedures for the milking equipment used for harvesting salable milk in Farm A consisted in a disinfection step before milking using peracetic acid, a main wash using an alkaline dairy detergent, and a final rinse using peracetic acid. On the other hand, Farm B used a disinfection step before milking with a chlorine disinfectant, and a main wash with an alkaline dairy detergent. Farm C did not use any disinfectant before milking, but a main wash with an alkaline detergent and then disinfection of milking equipment using a chlorinated dairy product. Both Farms B and C used an acid-rinse of milking equipment once per week.

The cows in Farms A, B, and C were milked twice a day. Milk collected from cows undergoing antibiotic treatments, diagnosed with clinical mastitis, or producing transition milk or colostrum was diverted, using the milking system vacuum and the individual milking units with attached milk tubes (milk hoses) connected to a milk can, to divert the waste milk away from the bulk tank.

### Microbiology and microscopy analysis

Milk hoses used to collect waste milk, transition milk, and colostrum on Farms A, B, and C were visually inspected to assess the presence of macroscopic adherences or films (i.e., visible films or adherences attached to surfaces) using a ~10.000 lx flashlight (9). The inner part of milk hoses showing macroscopic adherences was swabbed using sterile cotton swabs moistened with Neutralizing Buffer (Difco; BD Diagnostics, Sparks, MD) for microbiology analysis (6) at the Milk and Dairy Safety Laboratory, College of Veterinary Sciences, Universidad de Concepción, Chile. After swab sampling, the milk hoses were removed, replaced with new ones, and transported in a cooler with ice packs to the laboratory, where they were aseptically cut and processed for Scanning Electron Microscopy (SEM) and Confocal Spectral Microscopy (CSM) at the Spectroscopy and Electronic Microscopy Center (CESMI) and at the Advanced Microscopy Center (CMA Biobío) of the Universidad de Concepción, Chile, respectively.

Upon arrival to the Milk and Dairy Safety Laboratory, all swab samples were analyzed for the presence of *S. aureus, P. aeruginosa* and *Klebsiella* spp. For this purpose, swab samples were enriched in 10 mL Brain Heart Infusion broth for 48 h at 37°C (7, 9). After enrichment, 10 μL aliquots were streaked onto CHROMagar™ Staph. aureus (CHROMagar, France) (9), Cetrimide Agar (Merck, Darmstadt, Germany), and onto MacConkey agar (Oxoid, Basingstoke, UK) supplemented with 10 mg/L of ampicillin (11), for the isolation of *S. aureus, P. aeruginosa*, and *Klebsiella* spp., respectively. Putative *S. aureus* colonies (i.e., pink to mauve colonies), *P. aeruginosa* (i.e., yellow-green fluorescent colonies), and *Klebsiella* spp colonies (i.e., pink, dome-shaped mucoid colonies) were identified and transferred to a new plate for purification and storage at −80°C in Microbank Beads (Pro-Lab Diagnostics, Round Rock, TX) for further analysis. Subsequent confirmation of bacterial species was done by matrix-assisted laser desorption/ionization time-of-flight mass spectrometry (MALDI-TOF MS) MALDI Biotyper (Bruker Daltonics GmbH, Bremen, Germany) at Dr. Víctor Ríos Hospital in Los Angeles, Chile.

For microscopy analysis, the removed hoses were aseptically cut using scissors, as described by Latorre et al. (7). Staining and fixation of samples for SEM were done following the protocol described by Latorre et al. (9). Briefly, after staining, samples were put in 2.5% glutaraldehyde, washed 3 times with 1X Phosphate Buffered Saline (PBS) at room temperature, and then gradually dehydrated using graded series of ethanol. Samples were then stored at 4°C overnight and transported to CESMI for SEM analysis.

Samples prepared for CSM were also aseptically cut as described earlier, and then they were immersed in 4% paraformaldehyde at 4°C overnight for fixation before transportation to CMA for analysis. For CSM analysis, pieces of the hoses were incubated in the dark for 2 min in a Propidium Iodide (Sigma Aldrich, Darmstadt, Germany) solution prepared with PBS (1:500). After incubation, samples were washed 3 times with Phosphate-Buffered Saline and then mounted in a specimen support for scanning of the concave surface of the hoses where the adherences were attached. Images were obtained using a Confocal Spectral Microscope LSM 780 (Zeiss, Oberkochen, Germany) using a spectral scan at 405, 488, 543, and 633 nm with an objective Plan-Apochromat 40x.

Propidium Iodide staining was analyzed exciting the samples using 543 nm and scanning the *Z*-axis of the adherences attached to the hoses. In addition, a white-colored adherence in the milk hose sample collected from Farm A was analyzed using Nomarski microscopy and fluorescence was detected at 405 and 488 nm with an objective Plan-Apochromat 20x on a single confocal *Z* plane. Image reconstruction for all samples was done using the software Zen Lite (Zeiss, Oberkochen, Germany).

## Results

**Figure 1:**
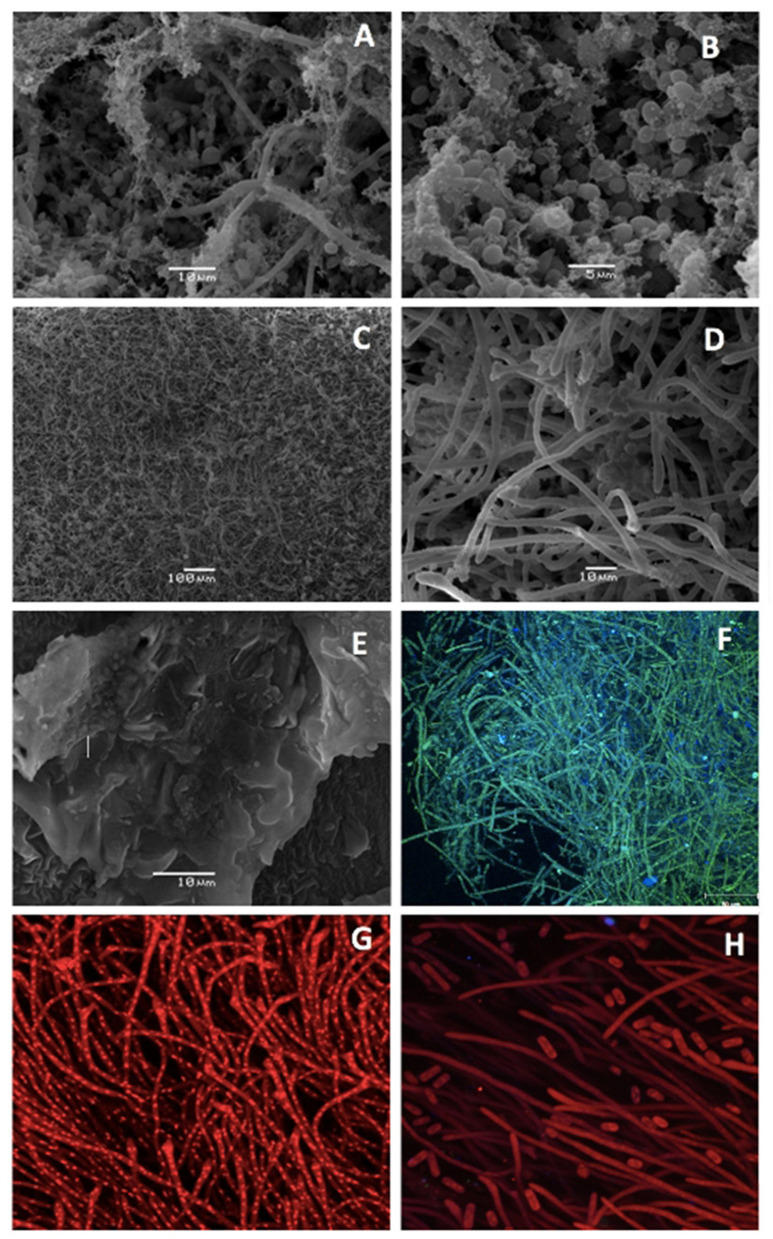
Scanning Electron Microscopy **(A–E)** and Confocal Spectral Microscopy **(F–H)** images of biofilms on the surface of milk hoses used to divert milk on three dairy farms: Farm A **(A,B,F)**, Farm B **(C,D,G,H)**, and Farm C **(E)**. Panel **(F)** shows a multispecies biofilm analyzed using Nomarsky microscopy (405/488 nm) and Panels **(F,G)** show a biofilm stained with Propidium Iodide (543 nm).

Both thick and thinner adherences were observed on the surfaces of all three milk hoses. All adherences were confirmed as microbial biofilms by microscopy analysis (Figure 1). Biofilms were composed of multispecies microbial communities, where associations between bacteria and other microorganisms within the biofilms were often observed. By SEM, the presence of cocci, rod-shaped bacteria, yeasts, and molds were observed on the samples (Figures 1A,B,D). In addition, exopolymeric matrixes, both in lax and compact shapes, were observed surrounding or anchoring the microbiota to the surface of the hoses. Biofilms on the surfaces of milk hoses from Farm A and B were abundant and fully covered the surface of the samples analyzed by SEM microscopy (Figures 1A–D); while on the milk hose collected from Farm C, the biofilms were present in a sparser fashion, resembling “patches” of compact multispecies biofilms (Figure 1E). In Farm A, differences were observed within the biofilm structure, where in areas closer to the surface (where milk circulates and a more expedite supply of nutrients and water exists) a greater abundance of yeasts and molds was observed, as compared to deeper areas of the biofilm where a predominance of bacteria was noted.

By CSM, on the milk hose sample from Farm A, it was possible to observe biofilms composed of long-green filamentous structures, as well as numerous blue structures of 1–2 μm. The filamentous structures may either correspond to mold hyphae or associations of numerous rod-shaped bacteria, while the blue structures correspond to bacteria (Figure 1F). In addition, biofilms positive to propidium iodide staining (Figures 1G,H) were observed on Farm B. Long filamentous, as well as smaller structures, both positive to the DNA markers used on these samples, were observed on this milk hose. Confocal Spectral Microscopy for the milk hose removed from farm C was not available due to malfunction of the microscope at the time of analyzing this sample.

In the microbiology analysis, combinations of *S. aureus, P. aeruginosa, K. pneumoniae, and K. oxytoca* were isolated from milk hose samples collected from Farms A and B (Table 1). From the milk hose sample collected from Farm C, none of the targeted bacteria were isolated, although bacterial growth was observed during the Brain Heart Infusion enrichment step.

**Table 1:** Characteristics of biofilms on milk hoses utilized to divert milk and colostrum on three Chilean dairy farms, evaluated by scanning electron microscopy (SEM), confocal spectral microscopy (CSM) analyses, and microbiological cultures.

**Milk hose origin**	**Sampled hoses/swabs**	**Biofilm SEM**	**Biofilm CSM**	**Biofilm's microbiological characteristics**	**Target bacteria^a^ isolated (*n*)**	**Use of milk hose: Divert milk/colostrum and transition milk collection**
Farm A	1/1	+	+	Multispecies biofilm (bacteria, Yeast, Molds)	•*S. aureus* (4) •*K. oxytoca* (3)	+/+
Farm B	1/1	+	+	Multispecies biofilm (bacteria, molds)	•*S. aureus* (1) •*K. pneumoniae* (3) •*P. aeruginosa* (3)	+/+
Farm C	1/1	+	N/A	Multispecies biofilm (bacteria)	N/D	+/+

a Target bacterial species were: *Staphylococcus aureus, Pseudomonas aeruginosa, Klebsiella* spp. (*n*) = number of putative colonies analyzed and confirmed on each sample.

N/A: confocal spectral microscopy was not available.

N/D: Growth of target bacterial species was not detected by microbiological culture analysis.

## Discussion

In Chile, as in many parts of the world, diverting transition milk and colostrum, or milk from cows that are suffering from clinical mastitis or undergoing antibiotic treatments, are common practices. As milk or colostrum deflected from the bulk tank is not sold to processors, the cleaning and sanitation procedures of the milking equipment used to collect this milk, are usually not part of the standard cleaning and sanitation procedures. On the farms analyzed in this study, waste milk, transition milk, and colostrum were collected using milk cans and a milk hose used only for this purpose. These milk cans for colostrum and waste milk (and their respective hoses) were not part of the standard cleaning and sanitation protocol in place for the milking equipment used to harvest salable milk on Farms A, B, and C.

Neglecting cleaning and sanitation protocols or inadequate washing of milking equipment, caused by insufficient temperature, chemical, and physical conditions (30) or even deficiencies in the flow of wash water (16), can cause the presence of milk residues in surfaces. These milk residues may aid the attachment of microorganisms acting as a conditioning film (23). These factors create ideal conditions for bacteria colonization and subsequent biofilm formation. The presence of biofilms in these milk hoses is relevant to cows and herd health because bacteria or other microorganisms can be sloughed from these biofilms as milk or colostrum passes through the hoses during milking, and biofilm microbiota can become part of the collected milk or colostrum. This waste milk, transition milk, and colostrum are often used to feed calves, exposing them to potentially harmful or pathogenic bacteria. For example, *S. aureus* was isolated from two of the three hoses which may have caused *S. aureus* exposure for calves fed this unpasteurized milk. Similarly, *Klebsiella* spp. were isolated from these same hoses, which may expose calves to this pathogen, which has been involved in respiratory infections (31–34) and meningoencephalitis (35) in bovines. Moreover, the third detected species, *Pseudomonas* spp., have also been implicated in respiratory (36), urinary tract (37), and otitis (38) in calves. Other microorganisms of importance for calves' health were not studied as they were beyond the scope of this research. Nevertheless, we cannot rule out the presence of other pathogens contained in the biofilms on these farms. Particularly, Farm A often reported morbidity and mortality of calves due to diarrhea and pneumonia. Although, these problems may have been triggered by other on-farm management practices, the role of pathogens in biofilms of milk hoses used to collect milk fed to calves cannot be ruled out.

Biofilms in milking equipment can cause milk contamination for long periods of time, therefore, the presence of biofilms in milking equipment used to divert milk from the bulk tank could represent a consistent, long-term exposure to pathogenic microorganisms for calves that are fed with waste milk or unpasteurized transition milk collected with this tainted equipment. In addition, the presence of bacterial pathogens in the herd environment, like the ones observed in biofilms from milk hoses on Farms A and B, cannot be disregarded. Previous reports have demonstrated that on-farm bacterial strains present in biofilms and bulk tank milk can also be found in the farm environment, including fecal samples from cows (15). Although the source or direction of pathogenic microorganisms' contamination could not be established in a previous study (15) the role of biofilms may play a relevant role as a source of on-farm pathogens. For example, at first calving, about 2–50% of heifers may present *S. aureus* intramammary infections. The role of flies and cross-suckling in calves have been reported as two plausible reasons to explain this rather high intramammary infection prevalence in heifers (39, 40). However, our findings suggest that biofilms may also play a role in the epidemiology of *S. aureus*-intramammary infections in heifers due to the potential transferring of pathogenic bacteria from biofilms to the oral epithelium of calves during feeding, and the subsequent contamination of teats of female calves due to cross-suckling. In addition, the presence of *Klebsiella* and *Pseudomonas*-containing biofilms could be a persistent source of these pathogens on dairy farms. These microorganisms could be then spread to the farm environment by different means such as fecal shedding (11) of calves fed with contaminated waste milk, handling of biofilm-contaminated milking equipment by dairy personnel, or even insects including flies. Therefore, more research on these topics is warranted to better understand the epidemiology of pathogenic microorganisms of importance in dairy herd health.

In addition, as milk from cows undergoing antibiotic treatments is also diverted from the bulk tank, bacteria contained in the biofilms of milking equipment used for this purpose are continually exposed to antibiotic residues contained in the diverted milk. This mechanism could be a source of risk for the development of antimicrobial resistant bacteria potentially contained in the biofilms. Eventually, these bacteria may pass to the milk used for animal feed and their environment. Penati et al. (41) reported diarrhea and changes in gut microbiome of calves that were fed with waste milk containing antibiotic residues. Therefore, limiting the exposure of calves to both pathogens and antibiotic residues that might be present in waste milk is of utmost importance on dairy farms to protect this group and the overall herd health.

From a public health perspective, although milk commercialized for human consumption must be pasteurized according to Chilean regulations that have been in place since 1930, there still exists an informal raw-milk market in Chile. Although no detailed data regarding the quantities of raw milk being sold, nor the milk quality or its source is available from these informal markets, it is unofficially known that some vendors may acquire diverted/waste milk from farms to be sold for human consumption through informal markets. This practice is usually done under the assumption that milk is always boiled before consumption, which may not necessarily be true in all cases, thus creating a public health risk due to the presence of pathogens in that milk.

Drinking raw milk is a common practice in many Latin American countries. This practice is mostly non-regulated and neither the sanitary status nor the origin of milk (whether bulk tank or discarded milk) are known. The public health risks associated to the potential presence of pathogens in raw milk (42, 43) is further compounded for the presence of potentially harmful bacteria from biofilms in milking equipment used to harvest waste milk, and also for the risk of antimicrobial resistant bacteria from this source.

Despite the fact that this study was carried out on only three farms, the specific practices observed on them are common among other dairy farms, particularly, the neglecting of cleaning and sanitation of milking equipment used to collect waste milk. The findings of this study are relevant for a better understanding of the on-farm persistence of pathogens involved in the epidemiology of diseases of the young stock and, ultimately, the whole herd.

We can conclude that hoses used to divert milk, transition milk or colostrum can be a source of biofilms and pathogenic bacteria. Therefore, the use of separate hoses to divert milk from cows undergoing antibiotic treatments or mastitis, and to collect colostrum or transition milk is warranted. Furthermore, cleaning and sanitation protocols of all surfaces in contact with milk or colostrum are crucial, as well as the pasteurization of transition and waste milk. In addition, waste milk used to feed calves constitutes not only a biosecurity issue and a source of calf pathogens, but also an increased exposure and threat for the whole herd health and, potentially, for human health.

## Data availability statement

The raw data supporting the conclusions of this article will be made available by the authors, without undue reservation.

## Ethics statement

No animals were used during this study and ethical considerations were reviewed and approved by the Institutional Bioethics and Biosafety Committee of the Universidad de Concepción, Chile.

## Author contributions

AL: field work, sample collection, microbiology analysis, sample fixation for microscopy, SEM visualization of samples, and preparation and drafting of the manuscript. MM: field work, sample collection, sample fixation for microscopy, and preparation and drafting of the manuscript. RO, JP, and AE: sample preparation, visualization, and image analysis by SEM. FN and KS: sample preparation, visualization, and image analysis by CSM. NG: sample preparation and analysis of isolates by MALDI-TOF MS. All authors have read, contributed to, and approved the final manuscript.

## Funding

Financial support was provided by the Chilean Commission for Scientific and Technological Research, FONDECYT Project No 11130343 (Santiago, Chile) and also financial support for open access publication fees were provided by Vicerrectoría de Investigaciǿn y Desarrollo (VRID), Universidad de Concepción-Chile.

## Conflict of interest

The authors declare that the research was conducted in the absence of any commercial or financial relationships that could be construed as a potential conflict of interest.

## Publisher's note

All claims expressed in this article are solely those of the authors and do not necessarily represent those of their affiliated organizations, or those of the publisher, the editors and the reviewers. Any product that may be evaluated in this article, or claim that may be made by its manufacturer, is not guaranteed or endorsed by the publisher.
